# Using organization theory to position middle-level managers as agents of evidence-based practice implementation

**DOI:** 10.1186/s13012-021-01106-2

**Published:** 2021-04-09

**Authors:** Sarah A. Birken, Graeme Currie

**Affiliations:** 1 Department of Implementation Science, Wake Forest School of Medicine, 525@Vine Room 5219, Medical Center Boulevard, Winston-Salem, NC 27157 USA; 2 Warwick Business School, University of Warwick, Coventry, CV4 7AL UK

**Keywords:** Middle-level managers, Organization theory, Evidence-based practice, Strategies, Implementation

## Abstract

Middle-level managers (MLMs; i.e., healthcare professionals who may fill roles including obtaining and diffusing information, adapting information and the intervention, mediating between strategy and day-to-day activities, and selling intervention implementation) have been identified as having significant influence on evidence-based practice (EBP) implementation. We argue that understanding whether and how MLMs influence EBP implementation is aided by drawing upon organization theory. Organization theories propose strategies for increasing MLMs’ opportunities to facilitate implementation by optimizing their appreciation of constructs which we argue have heretofore been treated separately to the detriment of understanding and facilitating implementation: EBPs, context, and implementation strategies. Specifically, organization theory encourages us to delineate different types of MLMs and consider how generalist and hybrid MLMs make different contributions to EBP implementation. Organization theories also suggest that MLMs’ understanding of context allows them to adapt EBPs to promote implementation and effectiveness; MLMs’ potential vertical linking pin role may be supported by increasing MLMs’ interactions with external environment, helping them to understand strategic pressures and opportunities; and how lateral connections among MLMs have the potential to optimize their contribution to EBP implementation as a collective force. We end with recommendations for practice and future research.

## Background

Evidence-based practice (EBP) implementation is influenced by middle-level managers (MLMs)—healthcare professionals who occupy roles including obtaining and diffusing information, adapting information and the intervention, mediating between strategy and day-to-day activities, and selling intervention implementation [1, 2]. MLMs include, for example, section chiefs, nurse managers, and team leaders [3, 4]. These “hybrid” managers not only implement strategies for EBP but inform these strategies in the first place [5]. Following which, we argue that MLMs’ potential to do so depends on key features of MLMs and their organizations; we can identify these key organization-level determinants of MLMs’ role in implementation using organization theory. In the text that follows, we offer insights from organization theory to help position MLMs as agents of EBP implementation. We also propose a research agenda to further advance understanding of MLMs’ role in EBP implementation.

## MLMs’ role in implementation

Elsewhere, we have argued that successful implementation requires harmonizing EBPs, the context in which they are to be implemented, and the strategies used to facilitate EBP implementation [6]. MLMs’ influence on EBP implementation requires a nuanced understanding and perspective on the role they play in shaping interactions among these dimensions. To date, many conceptualizations of MLMs’ role in implementation have focused too much on the EBP, the context, or implementation strategies [7–9]. Positioning MLMs as agents of implementation requires enhancing their relationship with all three.

Change interrupts normal patterns of organization and may be most effectively leveraged by those with a strategic position between those who develop strategy (e.g., top managers) and those who enact strategy (often frontline healthcare professionals) [1, 10]. MLMs are positioned to translate strategies into action. In the context of implementation, these respective roles often, but not always, mean that top managers make the decision to adopt EBPs; frontline healthcare professionals use EBPs, and MLMs facilitate EBP implementation. For example, executives in cancer programs accredited by the US Commission on Cancer identify the EBPs that they will use to address the accrediting organization’s standards [11]. In turn, MLMs are tasked with operationalizing the EBPs for integration into practice.

**Fig. 1:**
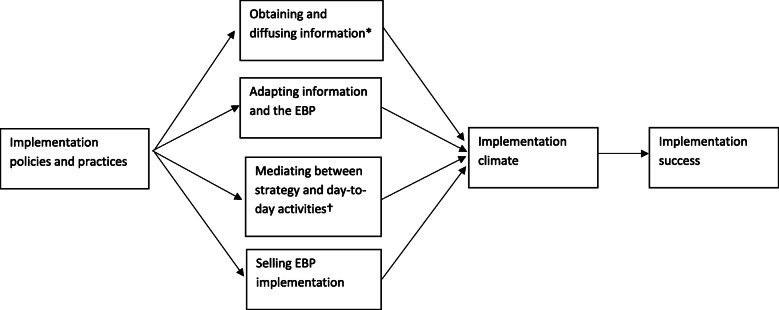
Refined theory of MLMs’ role in implementing EBPs in healthcare organizations [13]. MALA, middle-level manager; EBP, evidence-based practice; Asterisk indicates obtaining and diffusing information includes diffusing information internally and externally; dagger indicates mediating between strategy and day-to-day activities involves measuring performance and engaging in frontline activities

We posit that MLMs are best positioned to facilitate implementation with increased appreciation of EBPs, context, and strategies. Optimizing MLMs’ potential as agents of implementation may be achieved by modifying their capabilities, opportunities, and motivations [12]. Further, implementation policies and practices (i.e., implementation strategies) may enhance MLMs’ capability, opportunity, and motivation to carry out their hypothesized roles in implementation, in turn improving implementation climate (the extent to which implementation is expected, rewarded, and supported) and, subsequently, improving implementation success (see Fig. 1) [13]. Tool such as the Behavior Change Wheel are inadequate for addressing implementation determinants that lie at the organization level because the Behavior Change Wheel is rooted in the Theoretical Domains Framework, which is comprised of 33 theories of psychological theories that address individual-level determinants. To address organization-level determinants, organization theories are appropriate tools. Table 1 describes strategies that organization theories suggest may increase MLMs’ *opportunities* within contexts to facilitate implementation by optimizing their appreciation of EBPs, their context, and implementation strategies and ties them to MLMs’ hypothesized implementation roles.

**Table 1:** Organization theory propositions’ potential implications for middle-level managers

Theory	Proposition	Potential implications for MLMs’ use and selection of implementation strategies	Related MLM role
Contingency theory	The optimal structure of work is contingent on the uncertainty of the task and task environment: When uncertainty is higher, unprogrammed means of coordination will be the more effective way to structure a task; when uncertainty is low, programmed means of coordination will be more effective.	• Evaluate levels of uncertainty associated with implementation and its context. When uncertainty is high, avoid over-prescribing implementation strategies. • Evaluate levels of uncertainty associated with the implementation context. When uncertainty is high, limit efforts to tailor the context.	• Adapting information, the EBP, and implementation strategies
	Higher levels of interdependence (both within and between departments) will require greater investment in coordination (integration).	• Evaluate levels of interdependence required for implementation. For high levels of interdependence, invest resources in facilitating collaboration.	• Mediating between strategy and day-to-day activities
	The greater the differentiation among departments, the more difficult it will be to coordinate.	• Identify differences among departments and plan for their implications for implementation efforts.	• Mediating between strategy and day-to-day activities
Resource dependency theory	To acquire power, organizations exchange resources for dependence on other organizations within their field. That is, organizations want autonomy and/or control, but they need resources to survive and/or produce in a way that satisfies stakeholders’ demands.	• Contribute to the adoption decision considering its potential as a form of control—a source of legitimacy in the field, from the perspective of key stakeholders, and boon to the organization’s competitive edge^a^. • Compromise autonomy for all of the resources needed for implementation.	• Selling EBP implementation
	Competition increases uncertainty perceived by decision makers and decreases willingness to consider, adopt, or implement EBPs.	• Acknowledging above^a^, rigorously evaluate, appreciate, account, and plan for stakeholders’ resistance to EBP adoption and implementation.	• Selling EBP implementation
	Decreased munificence requires organizations to reduce their dependence on some resources and/or find alternative resources.	• In relatively under resourced organizations, acquire resources for implementation substitute resources with interorganizational partnerships (e.g., collaboratives).	• Obtaining and diffusing information and other resources
Complexity theory	Interdependencies contribute to sense making and self-organization.	• Create opportunities for and facilitate collaborative work among implementers.	• Mediating between strategy and day-to-day activities
	Interdependencies among people with diverse perspectives contribute to more effective sense making.	• Engage implementers who have diverse (clinical, cultural, etc.) perspectives. • Facilitate collaboration among implementers that elicits diverse perspectives.	• Mediating between strategy and day-to-day activities
	Feedback loops may amplify some effects and reduce others. At times, small changes will lead to large scale differences in outcomes (i.e., the butterfly effect) and vice versa.	• Monitor influences of changes over time. • Incorporate findings regarding changes’ influence into subsequent changes. • Monitor subsequent changes and repeat.	• Obtaining and diffusing information
	Change that is guided by minimum specifications allows individuals to self-organize most effectively.	• Build autonomy into implementers’ positions.	• Adapting information and the EBP
	The whole system is greater than the sum of its parts.	• Monitor processes and outcomes at organization and system levels.	• Obtaining and diffusing information

^a^*EBP* evidence-based practice

### Implementation strategies

In previous work, we conceptualized implementation strategies as implementation policies and procedures [14]. Klein and Sorra defined implementation policies and procedures as “the array of innovation, implementation, organizational, and managerial policies, practices, and characteristics that may influence innovation use” [12]. Increasingly, the implementation science literature uses frameworks to conceptualize strategies [15, 16]. Implementation researchers often aim to test discrete strategies or fixed combinations of strategies to promote implementation. For example, 140 trials have tested the effects of audit and feedback on quality of care [17]. In practice, however, it is uncommon for those tasked with implementation to use discrete or fixed implementation strategies. Instead, practitioners—including MLMs—iterate among whichever implementation strategies are available, potentially effective in facilitating implementation, or appealing for other reasons—practices that make understanding discrete strategies’ effectiveness challenging [18]. To increase MLMs’ potential to facilitate implementation, we must improve MLMs’ opportunity to select and use optimal implementation strategies—i.e., the strategies that are most likely to facilitate implementation.

Three organization theories are particularly useful in understanding how to improve MLMs’ opportunity to select and use optimal implementation strategies against the backdrop of their context and the EBP. Table 1 summarizes the organization theories described below along with potential implications for MLMs’ use and selection of implementation strategies and the related MLM implementation roles. First, contingency theory suggests that—broadly speaking—implementation strategies that focus on coordination are important when uncertainty is high in an organization. The process of implementation itself—i.e., integrating a new EBP into an organization—introduces uncertainty. Implementation may benefit from MLMs evaluating levels of uncertainty and adapting the EBP, context, and implementation strategies to account for degrees of uncertainty. For example, MLMs may perceive that, when uncertainty is high, over-prescribing implementation strategies may be counterproductive and that using implementation strategies that increase coordination, such as regular meetings and engaging key stakeholders, may be particularly productive when tasks are highly interdependent or when departments or units are highly differentiated. Employing these strategies is a way in which MLMs may mediate between strategy and day-to-day activities.

Second, resource dependency theory suggests that power (e.g., political capital, access to resources, market or social dominance) is accrued to organizations that can balance the dependence implied in acquiring necessary resources against the loss of power that dependence implies. In the context of selecting and using implementation strategies, MLMs face tradeoffs—expending power to acquire the resources necessary to implement EBPs. This is the case with external facilitation [19]. In contrast to external facilitators, who incur a cost to the implementing organization and are not embedded in an organization long-term, clinical champions’ efforts do not incur additional cost to the organization, and they are often permanent employees of the organization. Nevertheless, clinical champions often neither have formal training nor the resources that external facilitators have [20]. MLMs’ balancing of the resources-power tradeoff associated with implementation strategies may be facilitated by increasing their access to explicit information about the potential costs and benefits of available implementation strategies. Obtaining resources, including information, and diffusing them to key stakeholders (i.e., those who adopt and implement EBPs) may help middle managers to sell EBP adoption and implementation.

Third, complexity theory suggests that change occurs in complex adaptive systems (i.e., systems that are made up of many interdependent, heterogeneous parts that interact in a nonlinear fashion) [21, 22]. As such, MLMs’ influence on implementation may vary over time—perhaps with substantial influence in early stages of implementation and waning influence over time. Consistent with the temporal nature of MLMs’ influence on implementation, complexity theory suggests that MLMs’ selection of implementation strategies may be enhanced by considering implementation strategies’ relevance in a given stage of implementation—and of their organization’s history. For example, audit and feedback may be a particularly effective implementation strategy when an organization uses the strategy for other purposes (e.g., accreditation). In this example, the implementation strategy may be more effective if it is used in tandem with audit and feedback being used for accreditation purposes because it attends to features of the broader organizational context that are likely to interact with implementation. On the other hand, the implementation strategy is unlikely to be effective even if used in tandem with efforts elsewhere in the organization if it is pursued at an inopportune time (e.g., in the face of conflicting demands). MLMs can facilitate implementation by obtaining and diffusing information regarding the changes in the EBP, context, and implementation strategies and modifying them to mediate between strategy and day-to-day activities—for example, by facilitating collaboration among implementers with diverse perspectives.

## Synthesizing knowledge from organization theory to offer insight about middle managers’ role in implementation

Our review highlights that MLMs have potential to facilitate EBP implementation, should their opportunity for EBP implementation be enhanced. This requires their understanding of, and action around interaction of the EBP itself, the context into which EBP is to be implemented, and the array of implementation strategies at hand. As a general point, following this, we suggest that political attacks upon the value of MLMs in healthcare are misplaced. In the UK for example, politicians and media have attacked them as “men [sic’] in grey suits,” who do not add value and have engaged in successive rounds of their delayering [23]. In contrast, we argue that MLMs add value in terms of implementing EBPs when we support their opportunity to do so. Our key concern is how we foster opportunities among MLMs to facilitate EBP implementation. Derived from our discussion, we make the following assertions about enhancing opportunity for MLMs to implement EBPs.

First, considering the contingency of uncertainty and the corresponding importance of coordination of the actors necessary to support EBP implementation, we highlight the wide-ranging positioning of MLMs within an organization that allows them a role in relational coordination [24]. MLMs in hybrid roles that combine clinical and managerial responsibilities have deep relationships with their frontline clinical colleagues and can contribute their contextualized understanding to implement EBP [5]. The latter is crucial given contingency theory teaches us that MLMs are likely to *adapt* EBPs (and contexts and implementation strategies) as they adopt and implement EBPs. In short, if confined to an implementation role, that role is never likely confined to “mere” implementation of EBP that retains fidelity to its original evidence base [25]. Rather, MLMs will work to fit EBPs with context and available implementation strategies. On the one hand, this is necessary for EBP to “land” in context. On the other hand, EBP adoption may negatively or positively influence outcomes [26]. Further research might investigate the effects of MLM-driven adaptation of EBPs, contexts, and implementation strategies. Meanwhile, those generalist or pure play MLMs, most prone to criticism that they do not add value to healthcare, through their relationship with top managers, can draw down resources to support the scale-up of EBP [27]. Further research might more carefully delineate the respective contributions of different cadres of MLMs to EBP implementation, particularly given the lessons of complexity theory, which anticipates dynamism in EBPs, context, and implementation strategies; MLMs’ collective contributions may be best leveraged by accounting for this dynamism to bolster relational coordination in pursuit of EBP implementation, necessary because more formal structural coordination mechanisms commonly fall short when required to support change [24].

Second, above, we have highlighted the importance of MLM’s vertical linking pin role in the implementation of EBP; they foster relational coordination and translate EBP so it is sensitive to the context within which it is implemented. However, resource dependency theory suggests such a vertical linking pin role is no easy position to develop and maintain in the face of operational pressures to which MLMs must respond. Resource dependency theory suggests that, in the case of hybrid MLMs, the desire to maintain a close relationship with peers may mean that MLMs fail to take advantage of any opportunity for EBP implementation where their peers are resistant to any associated organizational change [28]. Our advice is to support hybrid MLMs that fulfill the role of a “tempered radical,” closely linked to their clinical peers, but also prepared to challenge well-established practice in pursuit of EBP [29]. The separation of executive decision-making around strategy development is also likely to constrain opportunities for MLMs to develop their linking pin relationships with those top managers who often develop strategy. To overcome this, MLMs might be included in strategic decision-making more, to engender the social networks and environmental understanding necessary to an effective EBP implementation role. Resource dependency theory suggests that understanding the environment, derived from which opportunity for resourcing might ensure, is enhanced through opportunities for MLMs to engage with external stakeholders; for example, those bodies commissioning and funding services or, where relevant, professional bodies. Further research might examine what interventions are effective to support MLMs in their linking pin role.

Finally, derived from complexity theory, we highlight the importance of lateral connections across managerial and professional organizations within the ranks of MLMs themselves, from which can be derived the opportunity for assimilation of their different knowledge components to inform EBP implementation from both a business and clinical perspective [30]. Different knowledge (e.g., management knowledge related to business case development for resourcing EBP and that related to clinical context) is relevant across different stages of EBP implementation [27]. The former is important as EBP implementation moves towards scaling up any intervention, while the latter is important during the development and implementation of EBP at a local level in the first place. We posit that MLMs should be regarded more than they have in the extant literature in terms of their influence by and on the collective—i.e., MLMs’ opportunity to influence implementation depends on collective-level phenomena (e.g., available implementation strategies), and MLMs’ influence on implementation falls at the collective level (i.e., implementation climate) [31]. We note that leadership development programs are proliferating to support MLMs enact their implementation role in healthcare that recognizes leadership as a collective or distributed phenomenon. This is government policy led in some instances (for example, see National Health Service Leadership Academy in England) and professional body led in other instances (for example, see Faculty of Medical Leadership and Management in England), there are local organization interventions [27]. Further research might examine the role of educational interventions in facilitating lateral connections across the MLM cadre and supporting identity transition towards EBP implementation.

In conclusion, following advocacy for health policymakers and practitioners to “dance” with organization theory [32], we have sought to provide insight for implementation scientists regarding the important role of MLMs derived from organization theory. In so doing, we have suggested prescriptions to enhance opportunity for MLMs to implement EBP that other implementation scientists may follow up in their empirical study.

## Data Availability

Not applicable

## Abbreviations

EBP: Evidence-based practice
MLM: Middle-level manager
