# Correction to: Using organization theory to position middle-level managers as agents of evidence-based practice implementation

**DOI:** 10.1186/s13012-021-01121-3

**Published:** 2021-04-21

**Authors:** Sarah A. Birken, Graeme Currie

**Affiliations:** 1 Department of Implementation Science, Wake Forest School of Medicine, 525@Vine Room 5219, Medical Center Boulevard, Winston-Salem, NC 27157 USA; 2 Warwick Business School, University of Warwick, Coventry, CV4 7AL UK

**Correction to: Implement Sci 16, 37 (2021)**

**https://doi.org/10.1186/s13012-021-01106-2**

Following publication of the original article [1], the authors reported a missing funding acknowledgement. The funding acknowledgement is given below, and the original article has been corrected.

This publication was supported in part by the Centers for Disease Control and Prevention of the U.S. Department of Health and Human Services (HHS) as part of a financial assistance award. The contents are those of the author(s) and do not necessarily represent the official views of, nor an endorsement, by CDC/HHS, or the U.S. Government.
